# Anti-Protozoal Activities of Cembrane-Type Diterpenes from Vietnamese Soft Corals

**DOI:** 10.3390/molecules200712459

**Published:** 2015-07-08

**Authors:** Nguyen Phuong Thao, Bui Thi Thuy Luyen, Reto Brun, Marcel Kaiser, Phan Van Kiem, Chau Van Minh, Thomas J. Schmidt, Jong Seong Kang, Young Ho Kim

**Affiliations:** 1 College of Pharmacy, Chungnam National University, Daejeon 305-764, Korea; E-Mails: thaonp@imbc.vast.vn (N.P.T.); luyenbthoaduoc@gmail.com (B.T.T.L.); 2 Institute of Marine Biochemistry (IMBC), Vietnam Academy of Science and Technology (VAST), 18-Hoang Quoc Viet, Caugiay, Hanoi 10000, Vietnam; E-Mails: phankiem@yahoo.com (P.V.K.); cvminh@vast.vn (C.V.M.); 3 Swiss Tropical and Public Health Institute (Swiss TPH), Socinstrasse 57, Basel CH-4002, Switzerland; E-Mails: reto.brun@unibas.ch (R.B.); marcel.kaiser@unibas.ch (M.K.); 4 University of Basel, Petersplatz 1, Basel CH-4003, Switzerland; 5 Institute of Pharmaceutical Biology and Phytochemistry (IPBP), University of Münster, PharmaCampus, Corrensstrasse 48, Münster D-48149, Germany

**Keywords:** soft coral, cembrane-type diterpenes, marine natural product, anti-protozoal activity, *Trypanosoma brucei*, *Leishmania donovani*, *Plasmodium falciparum*, neglected tropical diseases

## Abstract

Based on our previous finding that certain cembranoid diterpenes possess selective toxicity against protozoan pathogens of tropical diseases such as *Trypanosoma* and *Plasmodium*, we have subjected a series of 34 cembranes isolated from soft corals living in the Vietnamese sea to an *in vitro* screening for anti-protozoal activity against *Trypanosoma brucei rhodesiense* (*Tbr*), *T. cruzi* (*Tc*), *Leishmania donovani* (*Ld*), and *Plasmodium falciparum* (*Pf*). Twelve of the tested compounds displayed significant activity against at least one of the parasites. Specifically, 7*S*,8*S*-epoxy-1,3,11-cembratriene-16-oic methyl ester (**1**), (1*R*,4*R*,2*E*,7*E*,11*E*)-cembra-2,7,11-trien-4-ol (**2**), crassumol D (**12**), crassumol E (**13**), and (1*S*,2*E*,4*S*,6*E*,8*S*,11*S*)-2,6,12(20)-cembrantriene-4,8,11-triol (**16**) from *Lobophytum crassum*, *L. laevigatum*, and *Sinularia maxima* showed the highest level of inhibitory activity against *T. b. rhodesiense*, with IC_50_ values of about 1 µM or less. Lobocrasol A (**6**) and lobocrasol C (**8**) from *L. crassum* and *L. laevigatum* exhibited particularly significant inhibitory effects on *L. donovani* with IC_50_ values < 0.2 µM. The best antiplasmodial effect was exerted by laevigatol A (**10**), with an IC_50_ value of about 3.0 µM. The cytotoxicity of the active compounds on L6 rat skeletal myoblast cell was also assessed and found to be insignificant in all cases. This is the first report on anti-protozoal activity of these compounds, and points out the potential of the soft corals in discovery of new anti-protozoal lead compounds.

## 1. Introduction

Neglected tropical diseases (NTDs) are a group of seventeen mostly life threatening and/or disabling infections affecting more than a billion people worldwide. Most affected are poor populations in developing countries. People suffering from NTDs hence represent an unattractive market to private-sector research and development investment. Among the NTDs, infections caused by unicellular eukaryotic parasites (“protozoans”), *Trypanosoma brucei* (which causes human African trypanosomiasis (HAT) as well as Nagana affecting cattle), *T. cruzi* (causing Chagas disease), *Leishmania* spp. (causative agents for leishmaniases), most seriously demand the search for new effective, safe and affordable drugs. Malaria, caused by *Plasmodium* spp., is currently not classified as NTD but represents a major health threat to a large part of the world’s population. The search for new drugs against these diseases is an urgent need and natural sources such as marine organisms with their extremely diverse secondary metabolites may play an important role [1]. Nature is an attractive source of new therapeutic compounds, with a tremendous diversity found among millions of species of marine organisms, plants, and microorganisms. Intensive research over the last four decades has proven that marine organisms are magnificent sources of bioactive secondary metabolites [2]. To date no marine natural products and/or derivatives have entered preclinical development specifically for trypanosomatid diseases [3]. However, various marine natural products have been reported in the literature to possess anti-protozoan activity [4,5,6] but the full potential of marine organisms is still largely uninvestigated. Secondary metabolites produced by diverse marine organisms thus represent a huge repository of chemical structures for searching new drugs which can contribute to improved public health conditions in tropical developing countries [7,8].

**Figure 1:**
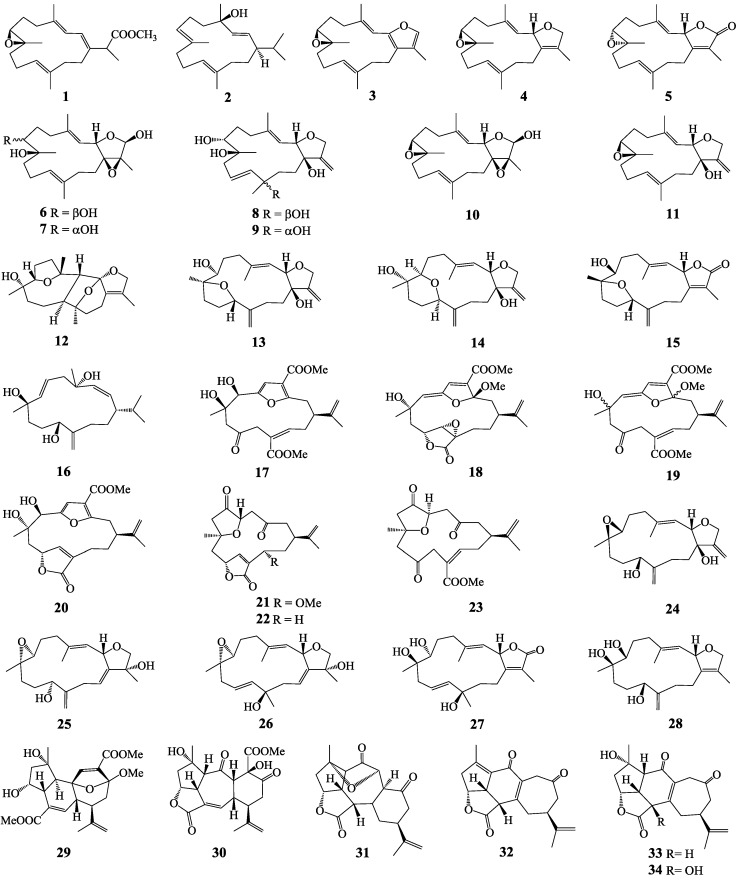
Chemical structures of cembrane-type diterpenes **1**–**34** isolated from Vietnamese soft corals.

In the course of our ongoing search for secondary metabolites with anti-protozoal activity from marine organisms [9], we recently reported on our study of methanolic extracts of the soft corals *Lobophytum crassum*, *L. laevigatum*, and *S. maxima* and some pure constituents isolated from them, which showed significant inhibitory *in vitro* activity against *T. brucei* (African sleeping sickness), with no toxicity on the mammalian HEK293T (human embryonic kidney) and HepG2 (human hepatoma) cell lines. Among the active compounds was a cembranoid diterpene, laevigatol B (**11**). Together with the previous observation that a plant-derived cembrane, serratol, isolated from *Boswellia serrata* (Burseraceae) also showed such activity [10], this finding prompted us to perform a more detailed study on the potential of this class of terpenoids against the mentioned diseases. We herein report on the results of *in vitro* activity tests of 34 cembranoid diterpenes (Figure 1), isolated as major pure constituents from Vietnamese soft corals, against the abovementioned protozoan parasites.

## 2. Results and Discussion

Thirty-four cembranoid diterpenes (for their structures see Figure 1), recently isolated from Vietnamese soft corals [11,12,13,14,15] were submitted to a multiple-target screening against *T. brucei rhodesiense* (*Tbr*), *T. cruzi* (*Tc*), *L. donovani* (*Ld*), and *P. falciparum* (*Pf*). Each compound was tested for growth inhibitory activity against each parasite at two concentrations, namely, 2.0 and 10.0 µM. The results are reported as supplementary information (Table S1). Quite noteworthy, *Tbr* appeared the most sensitive pathogen to the compounds under study most of which led to almost complete growth inhibition at 10.0 µM. In contrast, none of the compounds displayed any significant activity against the closely related *Tc*. Overall, twelve compounds (**1**, **2**, **6**, **8**, **10**, **12**, **13**, **15**–**18**, and **21**) showed a promising level of activity against at least one of the parasites, *i.e.*, >50% growth inhibition against *Tbr* at 2.0 µM and/or >50% inhibition at 10.0 µM against one of the other parasites. In case of such active compounds, consequently, IC_50_ values were determined. In addition, the cytotoxic activity of these compounds was investigated by determining their IC_50_ values of cell viability in the rat skeletal myoblast cell line L6 allowing the calculation of selectivity indices (SI = IC_50_(L6)/IC_50_(parasite)). The results are reported in Table 1.

**Table 1:** *In vitro* antiprotozoal activities and cytotoxic activity of marine metabolites.

Compounds	IC_50_ Values (μM) ^a^			
	*T. b. rhodesiense*	*L. donovani*	*P. falciparum*	Cytotoxicity L6
7 *S*,8*S*-Epoxy-1,3,11-cembratriene-16-oic methyl ester (**1**)	1.00 ± 0.19	ND	22.95 ± 0.15	>54
(1 *R*,4*R*,2*E*,7*E*,11*E*)-Cembra-2,7,11-trien-4-ol (**2**)	1.14 ± 0.50	ND	14.80 ± 0.90	49.40 ± 5.20
Lobocrasol A ( **6**)	9.97 ± 0.64	0.18 ± 0.02	ND	55.95 ± 3.65
Lobocrasol C ( **8**)	9.37 ± 0.74	0.17 ± 0.05	7.37 ± 0.35	40.45 ± 7.75
Laevigatol A ( **10**)	>20	ND	3.02 ± 0.50	28.75 ± 10.35
Crassumol D ( **12**)	0.61 ± 0.01	ND	ND	58.70 ± 1.00
Crassumol E ( **13**)	0.72 ± 0.04	ND	7.43 ± 0.86	43.00 ± 2.60
Crassumol G ( **15**)	12.20 ± 0.10	ND	7.57 ± 0.59	33.20 ± 13.50
(1 *S*,2*E*,4*S*,6*E*,8*S*,11*S*)-2,6,12(20)-Cembrantriene-4,8,11-triol (**16**)	0.65 ± 0.01	ND	ND	48.65 ± 3.45
Sinumaximol A ( **17**)	>20	ND	7.20 ± 0.71	45.00 ± 2.90
Sinumaximol C ( **18**)	9.88 ± 1.93	ND	ND	28.65 ± 9.95
13- *Epi*-scabrolide C (**21**)	11.76 ± 4.46	ND	ND	47.50 ± 6.00
Melarsoprol ^b^	0.005 ± 0.00	NT	NT	28.90 ± 9.30
Miltefosine ^b^	NT	0.14 ± 0.05	NT	143.10 ± 35.30
Chloroquine ^b^	NT	NT	0.004 ± 0.00	120.30 ± 27.50
Podophyllotoxin ^b^	NT	NT	NT	0.014 ± 0.002

^a^ All data represent the mean ± margin of deviation of two independent determinations; ^b^ Positive controls; ND: IC_50_ not determined due to low activity in initial screening; NT: not tested.

In case of *Ld*, only two compounds showed a high level of activity. Compounds **6** (lobocrasol A) and **8** (lobocrasol C) presented very low IC_50_ values of 0.18 and 0.17 µM, respectively, against this pathogen. This appears to be a highly specific antileishmanial effect since both compounds displayed only a low level of activity against *Tbr* and were neither particularly active against *Pf* nor significantly cytotoxic. It is also highly interesting to note that lobocrasol B (**7**), differing only in the configuration at C-7 (orientation of a single OH group) from lobocrasol A (**6**), was devoid of antileishmanial activity, which also points towards a very specific mechanism.

The IC_50_ values of seven compounds **1**, **2**, **8**, **10**, **13**, **15**, and **17** with antiplasmodial activity against *Pf* were generally much higher. Compound **10** (laevigatol A) with an IC_50_ of 3.0 µM was found to be the most active one in this series. It is thus the second cembranoid, besides serratol [10], reported to possess some activity against the malarial parasite. In the cytotoxicity assay on L6 cells laevigatol A (**10**) showed an IC_50_ value of 28.75 µM and its SI was 9.52 (Table 1).

The most active compounds displayed activity against *Tbr* ranging from 0.61 to 12.20 µM. The most promising activity was found with compounds **12** (crassumol D), **13** (crassumol E), and **16** ((1*S*,2*E*,4*S*,6*E*,8*S*,11*S*)-2,6,12(20)-cembrantriene-4,8,11-triol) displaying IC_50_ values of 0.61, 0.72, and 0.65 µM, respectively, followed by compounds **1** (7*S*,8*S*-epoxy-1,3,11-cembratriene-16-oic methyl ester) and **2** ((1*R*,4*R*,2*E*,7*E*,11*E*)-cembra-2,7,11-trien-4-ol) with IC_50_ values of 1.00 and 1.14 µM, respectively.

**Figure 2:**
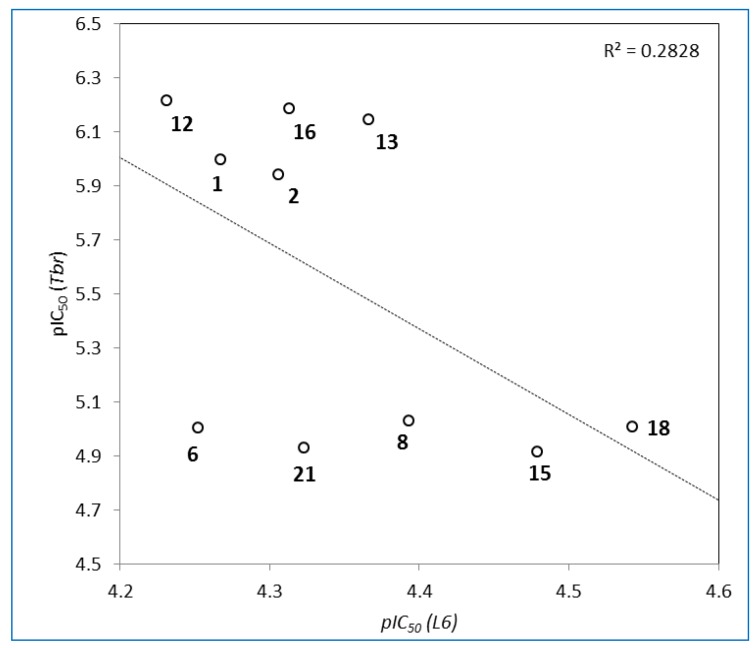
Plot of anti-trypanosomal activity *vs.* cytotoxicity as pIC_50_ [=−log (IC_50_)] data of compounds **1**, **2**, **6**, **8**, **12**, **13**, **15**, **16**, **18** and **21**.

Although the level of activity was generally lower than that of the positive controls, it is worth noting that their cytotoxity against the mammalian control cells was very low (IC_50_ values all > 28 µM), so that all these compounds show very favourable SI. The most favourable SI with respect to activity against *Tbr* were found in case of compounds **1**, **2**, **12**, **13**, and **16**, which were 54, 43, 96, 60, and 75 times more active against *Tbr* than against L6 cells, respectively. A plot of the logarithmic anti-*Tbr* activity data *vs.* the cytotoxicity data (Figure 2) for those compounds with an IC_50_(*Tbr*) < 20 µM clearly shows that there is no significant correlation between these activities and it may thus be expected that the antitrypanosomal effect is not due to general cytotoxicity but a specific and trypanosome-selective mechanism.

**Figure 3:**
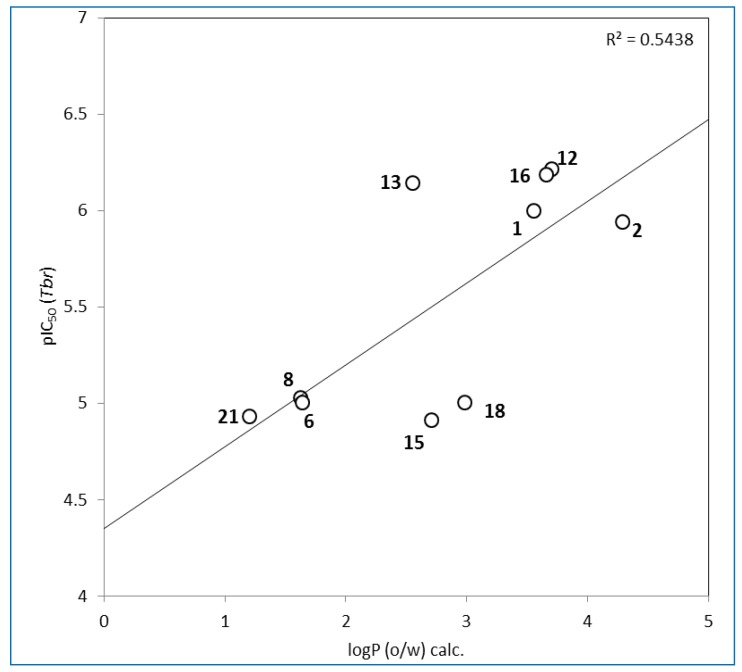
Plot of anti-trypanosomal activity as pIC_50_ [=−log (IC_50_)] *versus* calculated octanol/water partition coefficient data of compounds **1**, **2**, **6**, **8**, **12**, **13**, **15**, **16**, **18** and **21**.

Along the same lines, there is no clear correlation between the *in vitro* activities against the different parasites. This is much the same way as observed in other cases of other cembranoids reported to possess antiparasitic activity (such as serratol, laevigatol B, *etc.*) [9,10] and also points towards a specific activity towards *Tbr*. It appears difficult to recognize clear structure-activity relationships (SARs) among the present series. However, it is noteworthy that a clear correlation can be observed between the lipophilicity and anti-*Tbr* activity among the active compounds (Figure 3). Even though this correlation is not extremely strong, it appears obvious that a trend exists among these cembranoids to higher activity with increasing lipophilicity as would be expected in case that penetration of the compounds into the parasite plays a role. However, this correlation does not include the inactive compounds in the series, so that further factors must determine the general ability of such compounds to kill the parasites. First attempts to identify such structural properties by means of QSAR analyses using descriptors calculated with the software package MOE in a similar way as reported previously [16,17] were made but did not lead to satisfactory results so far. Further in-depth investigations in this direction are under way.

## 3. Experimental Section

### 3.1. Biological Material, Isolation and Structural Characterization of the Tested Compounds

The samples of soft corals (*Lobophytum crassum*, *L. laevigatum*, and *Sinularia maxima*) were collected at Khanhhoa (February 2009) and Thuathienhue (April 2010), Vietnam, identified by Do Cong Thung (Institute of Marine Environment and Resources, VAST). A list of these samples can be found in earlier publications [11,12,13,14,15]. Voucher specimens were deposited at the Institute of Marine Biochemistry and Institute of Marine Environment and Resources, VAST, Vietnam. Details on the isolation and structural characterization of the tested compounds were also reported in our earlier communications [11,12,13,14,15]. The purity of all compounds was >95% as assessed by high-performance liquid chromatography (HPLC) analysis (Shim-Pack Prep-ODS, waters ODS-3 column, 20 mm × 250 mm, 250 mm × 4.6 mm, 5 μm, 250 mm × 21.2 mm, 10 μm, *v*/*v*; Shimadzu, Kyoto, Japan); mobile phase: linear gradient of MeOH/H_2_O (50:50, 60:40, 78:22, *v*/*v*; flow rate: 1 mL/min, UV detection at 205, 210, 280, *v*/*v* nm).

### 3.2. Samples for Biological Tests

Stock solutions (concentration 10.0 mM) of the tested compounds were prepared in DMSO, kept at −20 °C, and diluted to the final concentration in fresh media before each experiment. In order to ensure unaffected cell growth, the final DMSO concentration did not exceed 0.5% in all experiments.

### 3.3. Anti-Trypanosomal Activity on T. brucei rhodesiense (Tbr)

Minimum Essential Medium with Earle’s salts (50.0 µL) supplemented with 0.2 mM 2-mercaptoethanol, 1.0 mM Na pyruvate and 15% heat-inactivated horse serum was added to each well of a 96-well microtiter plate. Serial drug dilutions of eleven 3-fold steps covering a range from 100 to 0.002 µM were prepared. Then 10^4^ bloodstream forms of *T. b. rhodesiense* STIB 900 in 50.0 µL of medium were added to each well and the plate incubated at 37 °C under a 5% CO_2_ atmosphere for 72 h. Alamar blue solution (10.0 µL of a solution of 12.5 mg resazurin dissolved in 100 mL distilled water) were then added to each well and incubation continued for a further 2–4 h. The plate was then read in a Spectramax Gemini XS microplate fluorometer (Molecular Devices Corporation, Sunnyvale, CA, USA) using an excitation wavelength of 536 nm and emission wavelength of 588 nm [18]. Fluorescence development was measured and expressed as percentage of the control. Data were transferred into the graphic programme Softmax Pro (Molecular Devices), which calculated IC_50_ values.

### 3.4. Antileishmanial Activity on L. donovani (Ld; Axenic Amastigote Assay)

Fifty µL of SM medium [19] at pH 5.4 supplemented with 10% heat-inactivated FBS, was added to each well of a 96-well microtiter plate. Serial drug dilutions were prepared covering a range from 100.0 to 0.002 µM. Then 10^5^ axenically grown *L. donovani* amastigotes (strain MHOM/ET/67/L82) in 50.0 µL medium were added to each well and the plate incubated at 37 °C under a 5% CO_2_ atmosphere for 72 h. Resazurin solution (10.0 µL, 12.5 mg resazurin dissolved in 100 mL distilled water) were then added to each well and incubation continued for a further 2–4 h. The plate was then read in a Spectramax Gemini XS microplate fluorometer (Molecular Devices, Sunnyvale, CA, USA) using an excitation wavelength of 536 nm and emission wavelength of 588 nm [18]. Fluorescence development was measured and expressed as percentage of the control. Data were transferred into the graphic programme Softmax Pro (Molecular Devices) which calculated IC_50_ values from the sigmoidal inhibition curves.

### 3.5. Antiplasmodial Activity on P. falciparum (Pf) NF54 Strain

Antiplasmodial activity was determined using the NF54 strain of *P. falciparum* (drug sensitive). A modification of the [^3^H]-hypoxanthine incorporation assay was used [20]. Briefly, infected human red blood cells (final parasitaemia and haematocrit were 0.3% and 1.25%, respectively) in RPMI 1640 medium with 5% Albumax were exposed to serial drug dilutions in microtiter plates. After 48 h of incubation at 37 °C in a reduced oxygen atmosphere, 0.5 μCi ^3^H-hypoxanthine was added to each well. Cultures were incubated for a further 24 h before they were harvested onto glass-fiber filters and washed with distilled water. The radioactivity was counted using a Betaplate™ liquid scintillation counter (Wallac, Zurich, Switzerland). The results were recorded as counts per minute (CPM) per well at each drug concentration and expressed as percentage of the untreated controls. From the sigmoidal inhibition curves IC_50_ values were calculated. Assays were run in duplicate and repeated once.

### 3.6. Cytotoxicity Assay against L6 Cells

Cytotoxicity was assessed using rat skeletal myoblasts (L6 cells) and a similar protocol as used for the *Tbr* assay. L6 cells were seeded in to RPMI 1640 medium supplemented with l-glutamine 2.0 mM, HEPES 5.95 g/L, NaHCO_3_ 2.0 g/L and 10% fetal bovine serum in 96-well microtiter plates (4000 cells/well). All following steps were according to the *T. b. rhodesiense* protocol (see Section 3.3. above).

### 3.7. Positive Controls

The compounds used as positive controls in the various bioassays were of commercial origin [16], with the exception of melarsoprol, which was a gift from WHO. Their purity (generally >95%) was specified by the manufacturers.

### 3.8. Statistical Analysis

The obtained data represent the mean ± margin of deviation of two independent experiments. The IC_50_ and CC_50_ (50% cytotoxic concentration) values were calculated using sigmoid dose-response curves performed using GraphPad Prism version 6.0 (GraphPad Software, San Diego, CA, USA), and the 95% confidence intervals (95% CI) were included. The ANOVA test was performed to evaluate the significance (*P* < 0.01) of data.

## 4. Conclusions

In conclusion, within a set of 34 tested cembranoid diterpenes from Vietnamese soft corals, we have identified twelve compounds with significant *in vitro* anti-protozoal activity. While cembranoid diterpenes **1**, **2**, **6**, **8**, **12**, **13**, **15**, **16**, **18**, and **21** showed activity against bloodstream forms of *T*. *brucei*, lobocrasol A (**6**) and lobocrasol C (**8**) were strongly and selectively active against *L. donovani*. Only one compound, laevigatol A (**10**) displayed antiplasmodial activity with an IC_50_ < 5.0 µM and can be considered moderately active. Since none of the compounds displayed any significant cytotoxicity, these and related compounds may open a new avenue for future drug development against African sleeping sickness and/or visceral leishmaniasis.

## Supplementary Materials

Supplementary materials can be accessed at: http://www.mdpi.com/1420-3049/20/07/12459/s1.

## References

[1] Schmidt T.J., Khalid S.A., Romanha A.J., Alves T.M.A., Biavatti M.W., Brun R., Costa F.B.D., Castro S.L.D., Ferreira V.F., Lacerda M.V.G.D. (2012). The potential of secondary metabolites from plants as drugs or leads against protozoan neglected diseases-Part II. Curr. Med. Chem..

[2] Blunt J.W., Copp B.R., Keyzers R.A., Munro M.H.G., Prinsep M.R. (2013). Marine natural products. Nat. Prod. Rep..

[3] Jones A.J., Grkovic T., Sykes M.L., Avery V.M. (2013). Trypanocidal activity of marine natural products. Mar. Drugs.

[4] Rubio B.K., Tenney K., Ang K.H., Abdulla M., Arkin M., McKerrow J.H., Crews P. (2009). The marine sponge *Diacarnus bismarckensis* as a source of peroxiterpene inhibitors of *Trypanosoma brucei*, the causative agent of sleeping sickness. J. Nat. Prod..

[5] Watts K.R., Tenney K., Crews P. (2010). The structural diversity and promise of antiparasitic marine invertebrate-derived small molecules. Curr. Opin. Biotechnol..

[6] Sanchez L.M., Knudsen G.M., Helbig C., Muylder G.D., Mascuch S.M., Mackey Z.B., Gerwick L., Clayton C., McKerrow J.H., Linington R.G. (2013). Examination of the mode of action of the almiramide family of natural products against the kinetoplastid parasite *Trypanosoma brucei*. J. Nat. Prod..

[7] Zofou D., Kang F.N., Sippl W., Efange S.M.N. (2013). Bioactive natural products derived from the Central Africanflora against neglected tropical diseases and HIV. Nat. Prod. Rep..

[8] Manilal A., Thajuddin N., Selvin J., Idhayadhulla A., Kumar R.S., Sujith S. (2011). *In vitro* larvicidal activity of marine algae against the human vectors *Culexquinque fasciatus* (Say) and *Aedes aegypti* (Linnaeus) (Diptera: Culicidae). Int. J. Zool. Res..

[9] Thao N.P., No J.H., Luyen B.T.T., Yang G., Byun S.Y., Goo J., Kim K.T., Cuong N.X., Nam N.H., Minh C.V. (2014). Secondary metabolites from Vietnamese marine invertebrates with activity against *Trypanosoma brucei* and *T. cruzi*. Molecules.

[10] Schmidt T.J., Kaiser M., Brun R. (2011). Complete structural assignment of serratol, a cembrane-type diterpene from *Boswellia serrata*, and evaluation of its antiprotozoal activity. Planta Med..

[11] Cuong N.X., Thao N.P., Luyen B.T.T., Ngan N.T.T., Thuy D.T.T., Song S.B., Nam N.H., Kiem P.V., Kim Y.H., Minh C.V. (2014). Cembranoid diterpenes from the soft coral *Lobophytum crassum* and their anti-inflammatory activities. Chem. Pharm. Bull..

[12] Thao N.P., Luyen B.T.T., Ngan N.T.T., Song S.B., Cuong N.X., Nam N.H., Kiem P.V., Kim Y.H., Minh C.V. (2014). New anti-inflammatory cembranoid diterpenoids from the Vietnamese soft coral *Lobophytum crassum*. Bioorg. Med. Chem. Lett..

[13] Quang T.H., Ha T.T., Minh C.V., Kiem P.V., Huong H.T., Ngan N.T.T., Nhiem N.X., Tung N.H., Tai B.H., Thuy D.T.T. (2011). Cytotoxic and anti-inflammatory cembranoids from the Vietnamese soft coral *Lobophytum laevigatum*. Bioorg. Med. Chem..

[14] Thao N.P., Nam N.H., Cuong N.X., Quang T.H., Tung P.T., Dat L.D., Chae D., Kim S., Koh Y.S., Kiem P.V. (2013). Anti-inflammatory norditerpenoids from the soft coral *Sinularia maxima*. Bioorg. Med. Chem. Lett..

[15] Thao N.P., Nam N.H., Cuong N.X., Quang T.H., Tung P.T., Tai B.H., Luyen B.T.T., Chae D., Kim S., Koh Y.S. (2012). Diterpenoids from the soft coral *Sinularia maxima* and their inhibitory effects on lipopolysaccharide-stimulated production of proinflammatory cytokines in bone marrow-derived dendritic cells. Chem. Pharm. Bull..

[16] Schmidt T.J., Nour A.M.M., Khalid S.A., Kaiser M., Brun R. (2009). Quantitative structure—Antiprotozoal activity relationships of sesquiterpene lactones. Molecules.

[17] Schmidt T.J., Costa F.B.D., Lopes N.P., Kaiser M., Brun R. (2014). *In silico* prediction and experimental evaluation of furanoheliangolide sesquiterpene lactones as potent agents against *Trypanosoma brucei rhodesiense*. Antimicrob. Agents Chemother..

[18] Räz B., Iten M., Grether-Bühler Y., Kaminsky R., Brun R. (1997). The Alamar Blue^®^ assay to determine drug sensitivity of African trypanosomes (*T.b. rhodesiense* and *T.b. gambiense*) *in vitro*. Acta Trop..

[19] Cunningham I. (1977). New culture medium for maintenance of tsetse tissues and growth of trypanosomatids. J. Protozool..

[20] Matile H., Pink J.R.L., Lefkovits I., Pernis B. (1990). *Plasmodium falciparum* malaria parasite cultures and their use in immunology. Immunological Methods.

