# Supplementary material for: Anti-Protozoal Activities of Cembrane-Type Diterpenes from Vietnamese Soft Corals
Source: Molecules. 2015 Jul 8;20(7):12459–68. doi: 10.3390/molecules200712459 (PMC6332397; doi:10.3390/molecules200712459)
Supplement: Supplementary file 1 [file molecules-20-12459-s001.pdf]

Supplementary Material

Anti-Protozoal Activities of Cembrane-Type Diterpenes from Vietnamese Soft Corals

Nguyen Phuong Thao [1,2], Bui Thi Thuy Luyen [1], Reto Brun [3,4], Marcel Kaiser [3,4], Phan Van Kiem [2], Chau Van Minh [2], Thomas J. Schmidt [5,*], Jong Seong Kang [1,*] and Young Ho Kim [1,*]

[1] College of Pharmacy, Chungnam National University, Daejeon 305-764, Korea;
E-Mails: thaonp@imbc.vast.vn (N.P.T.); luyenbthoaduoc@gmail.com (B.T.T.L.)

[2] Institute of Marine Biochemistry (IMBC), Vietnam Academy of Science and Technology (VAST), 18-Hoang Quoc Viet, Caugiay, Hanoi 10000, Vietnam; E-Mails: phankiem@yahoo.com (P.V.K.); cvminh@vast.vn (C.V.M.)

[3] Swiss Tropical and Public Health Institute (Swiss TPH), Socinstrasse 57, CH-4002, Basel, Switzerland; E-Mails: reto.brun@unibas.ch (R.B.); marcel.kaiser@unibas.ch (M.K.)

[4] University of Basel, Petersplatz 1, CH-4003 Basel, Switzerland.

[5] Institute of Pharmaceutical Biology and Phytochemistry (IPBP), University of Münster, PharmaCampus, Correnssstrasse 48, Münster D-48149, Germany;
E-Mail: thomschm@uni-muenster.de (T.J.S.)

* Author to whom correspondence should be addressed;
E-Mails: thomschm@uni-muenster.de (T.J.S.); kangjss@cnu.ac.kr (J.S.K.); yhk@cnu.ac.kr (Y.H.K.);
Tel.: +49-251-83-33378 (T.J.S.); +82-42-821-5928 (J.S.K.); +82-42-821-5933 (Y.H.K.);
Fax: +49-251-83-38341 (T.J.S.); +82-42-823-6566 (J.S.K. & Y.H.K.).

Table S1. Results of multiple-target screening of 34 cembranoid diterpenes against four protozoan pathogens.

Comps.	Cpd ID	% Growth Inhibition							
		T. b. rhodesiense		***T. cruzi***		***L. donovani***		***P. falciparum* NF54**	
		10.0 µM	2.0 µM	10.0 µM	2.0 µM	10.0 µM	2.0 µM	10.0 µM	2.0 µM
	**Ref.** [a]	99.4	100.0	103.8	75.6	96.3	97.2	99.9	100.1
	**Cembranoid Diterpenes**								
**1**	YHK-35A-LCR-7	95.5	25.4	30.0	19.7	12.2	24.2	98.9	0.0
**2**	YHK-35A-LCR-8	101.1	17.4	24.0	13.0	24.0	16.9	97.6	0.0
**3**	YHK-35A-LCR-1	94.3	20.8	32.8	22.6	21.1	27.8	78.3	0.0
**4**	YHK-35A-LCR-4	80.8	12.0	16.3	21.4	24.7	24.8	45.5	4.7
**5**	YHK-35A-LCR-6	101.0	30.5	30.2	27.9	19.9	25.0	0.0	0.0
**6**	YHK-35A-LCR-22	94.1	24.8	22.2	16.9	24.6	34.1	0.0	0.0
**7**	YHK-35A-LCR-21	102.7	102.6	27.1	14.3	66.3	66.9	26.5	4.0
**8**	YHK-35A-LCR-13	102.4	102.5	17.9	0.0	72.2	91.1	99.7	0.6
**9**	YHK-35A-LCR-30	93.4	29.4	17.6	0.0	19.5	25.4	0.0	0.0
**10**	YHK-35A-LCR-23	99.8	42.5	0.0	2.6	23.9	22.4	99.9	17.5
**11**	YHK-35A-LCR-24	94.5	21.0	0.0	0.0	16.8	20.5	84.9	0.0
**12**	YHK-35A-LCR-25	96.6	62.1	34.8	1.6	8.6	27.7	0.0	0.0
**13**	YHK-35A-LCR-26	60.9	15.3	13.5	0.0	18.9	14.0	0.0	0.0
**14**	YHK-35A-LCR-27	93.2	32.9	15.2	22.0	21.9	23.4	0.0	6.5
**15**	YHK-35A-LCR-29	96.8	31.3	23.2	18.4	21.6	26.0	87.4	0.0
**16**	YHK-35A-SM-14	95.8	60.8	19.2	12.7	18.8	21.9	0.0	0.0
**17**	YHK-35A-SM-21	93.3	27.6	0.0	0.0	23.4	26.3	92.1	43.2
**18**	YHK-35A-SM-22	96.3	67.0	24.2	0.0	32.3	22.5	11.3	0.0
**19**	YHK-35A-SM-27	93.6	25.4	28.4	20.1	0.0	2.7	0.0	0.0
**20**	YHK-35A-SM-18	89.6	27.0	22.9	25.3	3.1	0.0	0.0	0.0
**21**	YHK-35A-SM-24	102.4	93.0	29.1	12.7	20.0	22.3	35.1	0.0
**22**	YHK-35A-SM-30	90.6	26.4	13.8	0.0	21.6	23.2	0.0	0.0
**23**	YHK-35A-SM-26	91.5	26.2	26.0	12.2	11.5	28.1	0.0	0.0

Table S1. *Cont.*

Comps.	Cpd ID	*T. b. rhodesiense* 10.0 µM	*T. b. rhodesiense* 2.0 µM	*T. cruzi* 10.0 µM	*T. cruzi* 2.0 µM	*L. donovani* 10.0 µM	*L. donovani* 2.0 µM	*P. falciparum* NF54 10.0 µM	*P. falciparum* NF54 2.0 µM
	**Ref.** [a]	99.4	100.0	103.8	75.6	96.3	97.2	99.9	100.1
	**Cembranoid Diterpenes**								
**24**	YHK-35A-SM-4	82.5	11.0	28.1	30.0	12.1	28.1	0.0	0.0
**25**	YHK-35A-SM-5	73.7	10.6	24.6	0.0	0.0	6.8	0.0	0.0
**26**	YHK-35A-SM-8	86.5	3.8	23.1	19.5	24.8	29.2	0.0	0.0
**27**	YHK-35A-SM-1	90.8	23.4	19.9	18.9	21.2	25.4	0.0	0.0
**28**	YHK-35A-SM-13	96.3	40.0	27.1	3.8	23.1	23.3	4.2	0.0
**29**	YHK-35A-SM-16	95.0	23.1	26.9	24.0	15.1	20.6	6.2	0.0
**30**	YHK-35A-SM-19	92.3	24.1	0.0	6.3	19.8	22.6	0.0	0.0
**31**	YHK-35A-SM-23	92.3	23.4	17.3	9.9	19.6	30.7	3.9	0.0
**32**	YHK-35A-SM-29	93.9	42.6	3.1	7.4	24.9	23.8	7.9	0.0
**33**	YHK-35A-SM-17	22.7	6.7	21.9	19.1	11.6	37.1	0.0	0.0
**34**	YHK-35A-SM-20	102.3	2.6	0.0	11.1	47.4	25.0	0.0	0.0

(All values: % Growth Inhibition.)

[a] Reference compounds: *T. brucei rhodesiense*: Melarsoprol; *T. cruzi*: Benznidazole; *L. donovani*: Miltefosine; *P. falciparum*: Chloroquine.
